# A Novel Synaptic Vesicle Fusion Path in the Rat Cerebral Cortex: The “Saddle” Point Hypothesis

**DOI:** 10.1371/journal.pone.0100710

**Published:** 2014-06-24

**Authors:** Guido A. Zampighi, Raul Serrano, Julio L. Vergara

**Affiliations:** 1 Department of Neurobiology, UCLA School of Medicine, University of California Los Angeles, Los Angeles, California, United States of America; 2 Department of Physiology, UCLA School of Medicine, University of California Los Angeles, Los Angeles, California, United States of America; 3 Jules Stein Eye Research Institute, UCLA School of Medicine, University of California Los Angeles, Los Angeles, California, United States of America; Duke University Medical Center, United States of America

## Abstract

We improved freeze-fracture electron microscopy to study synapses in the neuropil of the rat cerebral cortex at ∼2 nm resolution and in three-dimensions. In the pre-synaptic axon, we found that “rods” assembled from short filaments protruding from the vesicle and the plasma membrane connects synaptic vesicles to the membrane of the active zone. We equated these “connector rods” to protein complexes involved in “docking” and “priming” vesicles to the active zone. Depending on their orientation, the “rods” define two synaptic vesicle-fusion paths: When parallel to the plasma membrane, the vesicles hemi-fuse anywhere (“randomly”) in the active zone following the conventional path anticipated by the SNARE hypothesis. When perpendicular to the plasma membrane, the vesicles hemi-fuse at the base of sharp crooks, called “indentations,” that are spaced 75–85 nm center-to-center, arranged in files and contained within gutters. They result from primary and secondary membrane curvatures that intersect at stationary inflection (“saddle”) points. Computer simulations indicate that this novel vesicle-fusion path evokes neurotransmitter concentration domains on the post-synaptic spine that are wider, shallower, and that reach higher *average concentrations* than the more conventional vesicle fusion path. In the post-synaptic spine, large (∼9× ∼15 nm) rectangular particles at densities of 72±10/ µm^2^ (170–240/spine) match the envelopes of the homotetrameric GluR2 AMPA-sensitive receptor. While these putative receptors join clusters, called the “post-synaptic domains,” the overwhelming majority of the rectangular particles formed bands in the “non-synaptic” plasma membrane of the spine. In conclusion, in the neuropil of the rat cerebral cortex, curvatures of the plasma membrane define a novel vesicle-fusion path that preconditions specific regions of the active zone for neurotransmitter release. We hypothesize that a change in the hybridization of the R-SNARE synaptobrevin from parallel to antiparallel swings the synapse into this novel vesicle-fusion path.

## Introduction

Over fifty years ago, a series of groundbreaking studies using electron microscopy revealed what is currently known about the structure of synapses in the nerve systems of vertebrates and invertebrates [1]–[7]. The structure that emerged from these studies was an asymmetric junction between terminals from neurons that are separated by sizable extra-cellular spaces (∼30 nm width). Since transmission at synapses is unidirectional, the terminals from these neurons were referred as pre- and post-synaptic. The pre-synaptic terminal contains clusters of vesicles (∼45 nm diameter) that fuse at specialized plasma membrane regions, called “active zones” [8]. Associated with the active zones these early studies identified “pre-synaptic grids” [2]–[3] that face the neurotransmitter receptor molecules in the post-synaptic terminal. The structure of CNS synapses was entirely consistent with the conclusion, based upon studies of the neuromuscular junction [9], that neurotransmitter release occurs in unitary packages, “quanta,” when a vesicle fuses with the membrane of the active zone. In essence, synapses align the quanta released by the pre-synaptic to the location of the neurotransmitter receptors in the post-synaptic neuron across the extra-cellular cleft.

What makes neuronal exocytosis special is the speed with which vesicles fuse after calcium ions enter the pre-synaptic terminal [10]. This property stems from the pre-conditioning of vesicles (e.g. “docking” and priming”), which involves the assembly of complexes of Q- and R-SNAREs proteins (syntaxin-1, SNAP-25 and synaptobrevin) [11], and accessory proteins that include Rab, Sec1/Munc18, CATCHR tethering and proteins that convey calcium sensitivity to the complex (synaptotagmin and complexin) [12]–[14]. It is accepted that the parallel hybridization of the alpha helices of the Q- and R-SNARES in the *trans-* configuration guides a small pool of synaptic vesicles (called “readily releasable”) into physical contact with the membrane of the active zone. While the kinetics, release probabilities, and calcium-dependence of the “readily releasable” pool have been characterized [15]–[17], the structural properties that distinguish this pool from vesicles in the “reserve pool” remain unsettled.

In cultured hippocampal neurons, imaging of fluorescently labeled proteins has been instrumental in parsing the individual contributions of proteins involved in the exocytotic pathway [18]. In brain tissues where the storage and processing of information in fact occur, labeling of specific proteins is difficult because synapses are clustered at high densities in regions called “neuropil” [7]. An additional limitation is the large number of proteins involved in the neuronal exocytotic pathway. For example, a single synaptic vesicle (surface area ∼6×10^−3^ µm^2^) comprises over 100 copies of twelve different proteins [19]; a even larger number of proteins has been reported for the active zone [20]. We hypothesize that a better understanding of synaptic transmission can be attained after defining the constraints that the protein assemblies introduce in synapses where information storage and processing does occur.

In this study, we examined the structural organization of the synaptic neuropil in the rat cerebral cortex at ∼2 nm resolutions and in three-dimensions, using freeze-fracture methods developed in our laboratory [21]–[24]. We found that in the cortical pre-synaptic axon, “rods” connecting vesicles to the plasma membrane likely represent trans-SNARE complexes. When these “rods” are parallel to the plasma membrane, the vesicles hemi-fuse apparently anywhere (“randomly”) on flat regions of the active zone; this is in agreement with the SNARE hypothesis [12]–[14]. Nevertheless, when the “connector rods” are perpendicular to the plasma membrane (as frequently occurs) the synaptic vesicles hemi-fuse at the base of “indentations” which are spaced 75–85 nm center-to-center, arranged in files, and contained within gutters. The spacing of the vesicle/indentation complexes ensues from curvatures of the plasma membrane that intersects at stationary inflection (“saddle”) points. Computer simulations indicate that synaptic vesicles that fuse within the gutters create neurotransmitter domains that are wider, shallower and reach higher *average concentrations* than when they open on flat regions of the membrane of the active zone. We conclude that the formation of stationary “saddle” points define a novel vesicle-fusion path that is a permanent component of synaptic transmission in the neuropil of the rat cerebral cortex.

## Results

### A. Neuropils and Synapses

**Figure 1:**
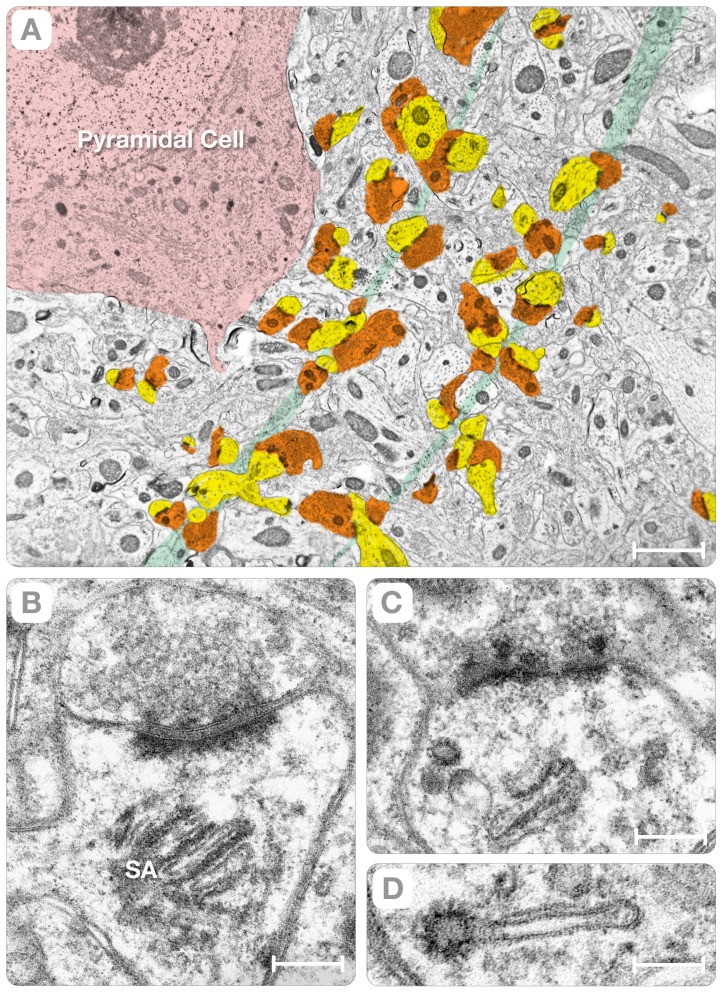
The Neuropil. Panel **A** is a low magnification view of a neuropil in the rat cerebral cortex. The thin section exposed the soma of a pyramidal cell (red) and the distribution of axons, dendrites and dendritic spines. To highlight the arrangement of synapses in parallel bands that are spaced ∼4 µm apart, the pre-synaptic axon terminals were colored in *orange* and the post-synaptic spines in *yellow*. Panels **B & C** are higher magnification views of individual synapses. The axon terminal has clusters of synaptic vesicles (40–50 nm diameter) and a specialized region, called the “active zone.” A distinctive property of cortical synapses is the existence of electron-dense particles arranged in the “pre-synaptic grid” [31] and layers of electron dense material at the PSDs. A unique organelle, called the “spine apparatus” (SA, **B**), occupies the neck of the spine. Other organelles include “coated” vesicles and a complex cytoskeleton comprised of thin filaments and dense particles (the post synaptic web). Panel **D** shows a “coated” vesicle at the end of a flatten cistern of the spine apparatus. Bars: A = 1.8 µm, B = 0.2 µm, C = 0.3 µm and D = 0.1 µm.

In the cerebral cortex, synapses between interneurons occur at high density in specialized regions, called neuropil, where the incoming axons lose their myelin sheets and projections from astrocyte are scarce (Palay and Chan-Palay, 1976) (Arellano et al., 2007). The pre-synaptic terminals are axons from neurons in the thalamus and cortical regions (orange, Fig. 1A) and the post-synaptic terminals are dendritic spines from pyramidal cells (yellow, Fig. 1A). A cursory low magnification analysis of the cortical neuropil suggests an indiscriminate arrangement of synapses. Yet, the apparent lack of order results from the partial sampling of the terminal axons and the dendritic spines in thin sections (∼50–80 nm) and projecting the entire thickness of the sections onto single planes (the “projection” artifact) [25]. When this limitation is understood, sections of the neuropil cut in orthogonal directions reveals that the synapses are in fact arranged in parallel bands spaced ∼4 µm apart (green lines, Fig. 1A). We construed this long-range order of synapses to be a reflection of the helical arrangement of dendritic spines first recognized in Purkinje cells of fish and mouse [26].

In the neuropil, the dendritic spines are quasi-cylinders that measure 0.8–1.3 µm in length and 0.6–0.8 µm (n = 41) in diameter. Some spines terminate as ellipses with semi-axes a = 0.8±0.15 µm and b = 0.450.1 µm (n = 9; yellow, Fig. 1A). The principal organelle of the spines is the cytoskeleton (i.e. the “post-synaptic web”). The enigmatic “spine apparatus” [2], comprised of a variable number of flattened cisterns that are interspaced with layers of electron-dense material (SA, Fig. 1B), is located at the neck of the spine. There are also “coated” vesicles (90–110 nm diameter) located at the ends of the cisterns of the apparatus (Fig. 1C and D). These morphological properties are consistent with post-synaptic spines undergoing active trafficking of molecules to and from the plasma membrane.

**Figure 2:**
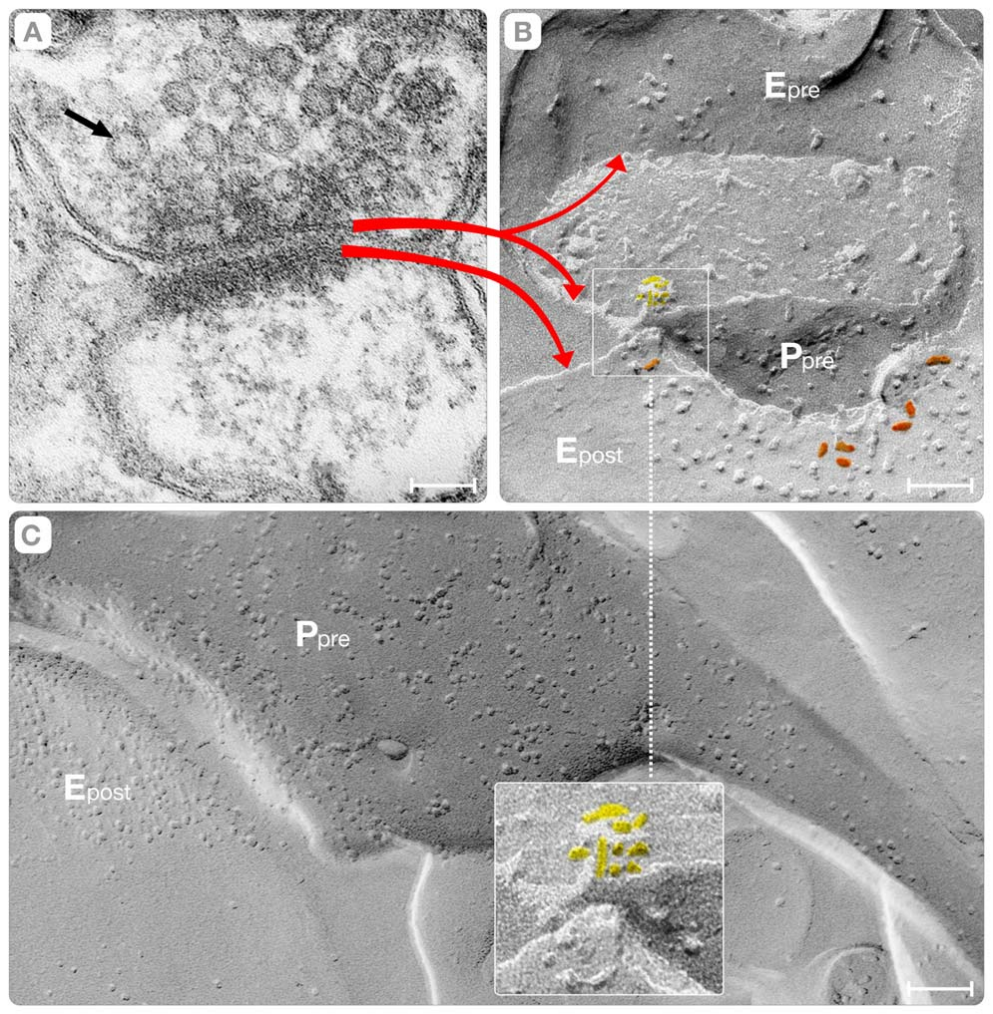
Fracturing the Synapse. Panels **A–B** show synapses from the cortical neuropil prepared by thin sectioning (A) and freeze-fracture (B) electron microscopy. The red arrows correlate the plasma membranes of both terminals with their respective fracture faces. In thin sectioning, the membranes exhibit tri-layer appearances resulting from the arrangement of the phospholipids in bilayer structures. This bilayer structure is also discernible in the synaptic vesicles (black arrow, **A**). The membrane of the active zone splits into complementary protoplasmic (P_pre_) and external (E_pre_) fracture faces (upper red arrow). Because of geometric constraints, only the external face (E_post_) of the plasma membrane, characterized by a cluster of intra-membranous particles, is observed (lower red arrow). Within the cluster, a subset of particles exhibited a rectangular shape (orange) that fit the envelope of the structure of the AMPA-sensitive receptor [30]. The synaptic vesicles appear as hemispheres associated to the P or E face of the active zone (square). Panel **C** shows the transition of a cylindrical axon into the enlarged varicosity (P_pre_) that synapses with a post-synaptic spine (E_post_). The inset shows the synaptic vesicle associated with the active zone (panel **B**, P_pre_), at higher magnification. The intra-membranous particles (yellow) highlight the asymmetric distribution of proteins of vesicles associated with the active zone. Bars: **A–B** = 70 nm, **C** = 100 nm.

When entering the neuropil, the pre-synaptic axon enlarges into varicosities or “boutons” [27]. This process transforms the 0.15–0.2 µm diameter axons into rectangular parallelepipeds (boxes) with axes a = 1.2±0.25 µm, b = 0.6±0.1 µm and c = 0.3±0.05 µm (n = 55). This cylinder-to-box transformation (Fig. 2C) has several consequences. First, it increases the terminal's volume (∼11-fold) and surface area (∼6-fold) while decreasing the S/V ratio from ∼28 µm^−1^ to ∼12.5 µm^−1^ (i.e. enhancing stability). Second, it compels the active zone to adopt rectangular shapes with axes measuring a = 0.4 to 0.67 µm and b = 0.12 to 0.2 µm (n = 31). Third, it forces the synapses in the region into parallel bands that are spaced ∼4 µm apart (green bands, Fig. 1A). The confluence of specialized organelles, such as the pre-synaptic grid, the “coated” vesicles, and the spine apparatus, bolster the idea of synaptic strength.

### B. Synaptic Proteins

**Figure 3:**
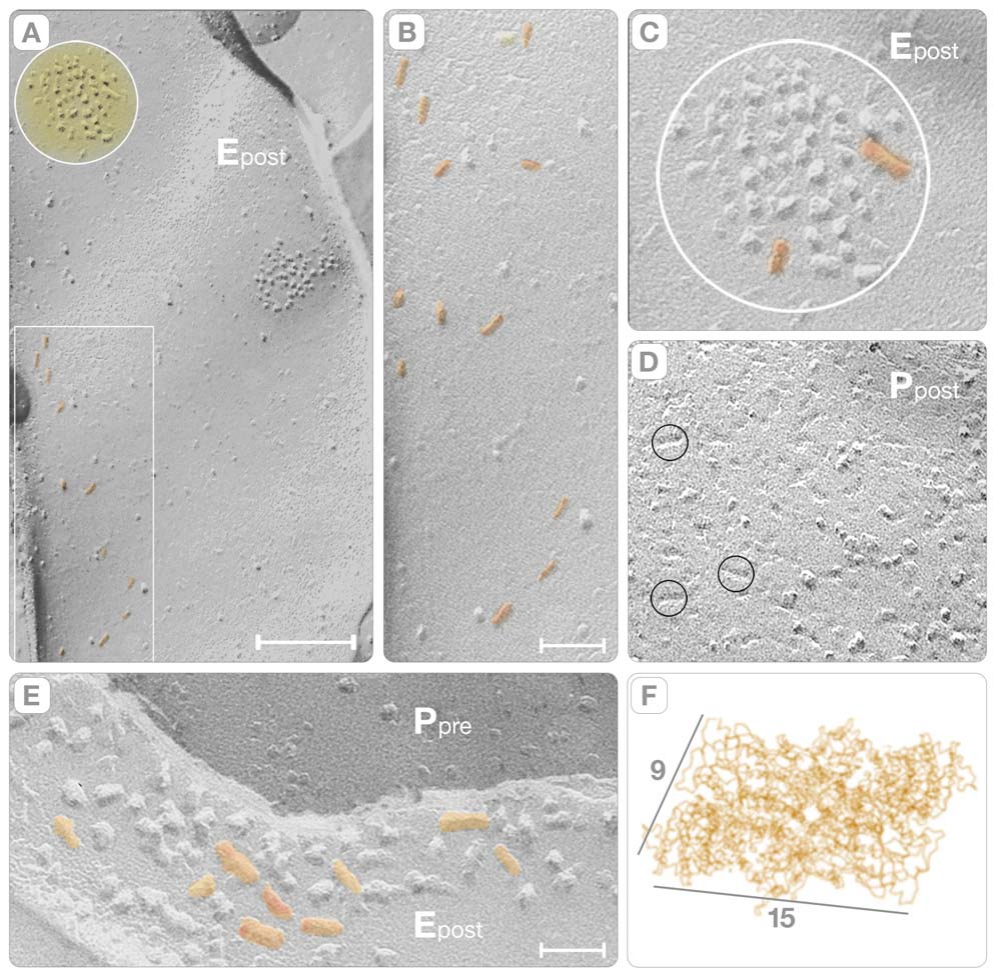
Fracture-Faces of Dendritic Spines. Panel **A** shows the E face of the plasma membrane of a dendritric spine (E_post_). The principal characteristics of the spine are: a) a low density of intra-membranous particles; and, b) the clusters comprising the PSDs (yellow circle). Individual rectangular particles (15×9 nm) extend alongside bands (rectangle) on the “non-synaptic” plasma membrane or join the clusters at the PSDs. Panel **B** is a higher magnification view of the region inside the rectangle to highlight the distribution of intra-membranous particles (colored orange). Panel **C** is a higher magnification view of the PSD in panel **A**. Only two rectangular particles (orange) are present in this particular cluster. Panel **D** shows the complementary P face (P_post_) of the PSD to show the “reverse” fracture pattern of the intrinsic proteins. Three small circles highlight elongated pits in this face, representing putative neurotransmitter receptors. Panel **E** is a higher magnification view of a PSD (E_post_). In contrast with the PSD in panel **C**, this one contains putative receptors (some of which are colored in orange). Bars: A = 0.1 µm, B = 50 nm, C–E = 20 nm. Panel **F** is a view of the homotetrameric AMPA-sensitive receptors from the x-ray diffraction model (Sobolevsky et al., 2009). Note that the dimensions of the rectangular particles in the clusters (orange) match the envelope of the model.

**Figure 4:**
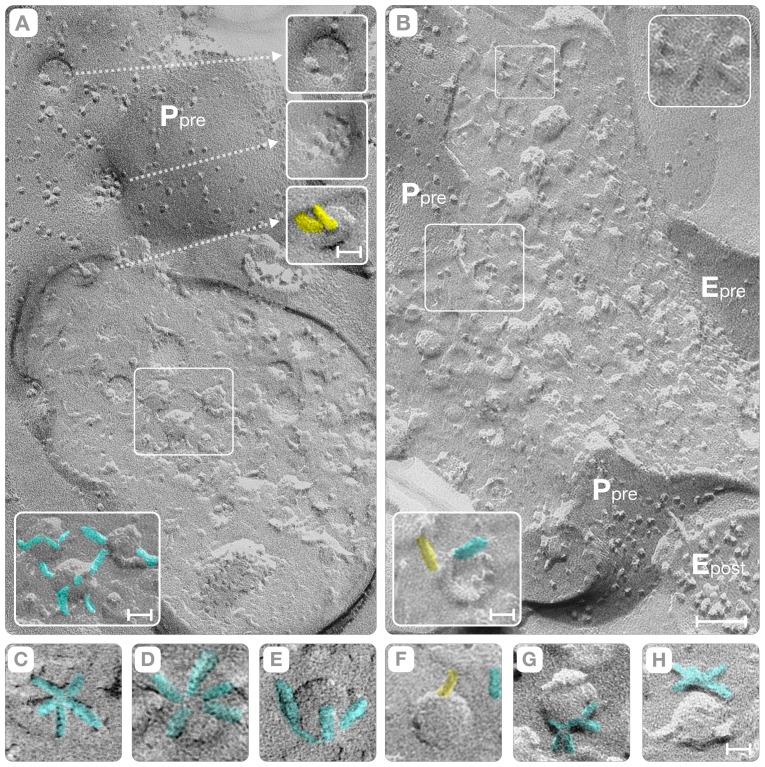
Fracture-Faces of Pre-Synaptic Terminals. Panel **A** shows two pre-synaptic axon terminals (“boutons”) identified from the synaptic vesicles in the cytoplasm and the protoplasmic fracture faces of the active zone (P_pre_ and E_pre_). The synaptic vesicles in the cytoplasm (“reserve” pool) appear as concave and convex hemispheres interconnected by short filaments (colored cyan in the lower inset). Panel **B** includes a small region area of the PSD (E_post_). In both **A** and **B** panels, “rods” connect synaptic vesicles to the active zone (yellow, third upper lower inset in **A** and lower inset in **B**). Depressions, called indentations, mark the location where synaptic vesicles dock or fuse with the membrane of the active zone (P_pre_, **A**). One indentation has a cluster of particles (middle inset, **A**) while others have smother walls with fewer particles (upper inset in **A**). Panels **C–E** and **G–H** show orthogonal views of the proteins on the surface of individual synaptic vesicles (cyan). Panels **C–D** show top views of two vesicles of the “reserve” pool, respectively, in which the fracture plane exposed their “true” (un-fractured) surface. In these vesicles, five filaments (cyan) occupy one of their poles. Panel **E** shows a bottom view, and panels **G–H** show side views, of vesicles in order to further confirm their polar distribution. Panel **F**, and the inset of panel **B** above, shows that the “rods” (yellow) connect both the convex and the concave hemispheres of synaptic vesicles to the plasma membrane. Bars: Panels **A–B** = 0.1 µm; insets in panel **A** = 25 nm; Insets in panel **B** = 18 nm; Panels **C–H** = 20 nm.

We used freeze-fracture methods to characterize the proteins of synaptic vesicles, the pre- and post-synaptic plasma membranes, and the protein assemblies comprising the vesicle/plasma membrane interface. The advantage of freeze-fracture is that membranes frozen at liquid nitrogen temperatures fracture down the middle of the phospholipid bilayers into complementary protoplasmic (P) and external (E) faces (red arrow, Fig. 2A–B). Integral membrane proteins appear as “intra-membranous” particles of different dimensions and shapes and complementary pits on these faces [28]–[29]. In the cortical neuropil, the fracturing process yielded eight fracture faces from the pre-synaptic axon (the “active zone” and the “non-synaptic” plasma membrane), the synaptic vesicles (concave and convex) and the post-synaptic spine the astrocyte's plasma membrane (not shown). After identifying these ten faces, we focused on the fracture faces from the active zone (P_pre_ and E_pre_), the post-synaptic spine (P_post_ and E_post_) and the concave and convex hemispheres of the synaptic vesicles (Figs. 2B, 3A–E and 4A–C). We reasoned that analysis of the connections between these six faces would reveal consensus structures of the vesicle/plasma membrane interface at the active zone.

In the pre-synaptic axon, the plasma membrane fractures asymmetrically leaving 1,113±110 particles/ µm^2^ (mean ±SD; n = 22) on the P face and 200–300 particles/ µm^2^ on the E face, which are comparable to those of Streit et al., 1972. The active zone was discerned by the presence of depressions on the P face and by complementary elevations on the E face that mark the places where synaptic vesicles first associate, and later fuse, with the plasma membrane (P_pre_, Fig. 4A). The membrane of a post-synaptic spine was identifiable because intrinsic proteins formed clusters of intra-membranous particles on the E face and complementary pits on the P face. These clusters are apposed to the location of the active zone in the pre-synaptic axon (E_post_, Figs. 2B, 3B, C–E; P_post_, Fig. 3D). We took advantage of this “reverse” fracture pattern of the E face clusters to identify those particles that correspond to neurotransmitter receptors in the plasma membrane of the dendritic spine. The E face clusters varied in area (0.011–0.3 µm^2^), and their total number of intra-membranous particles (30–180). This variability equated to a wide diversity of densities (from ∼1,560/ µm^2^ to ∼3,000/ µm^2^). Within the E face cluster, the intra-membranous particles exhibited “signatures” (i.e., dimensions and shapes) that embodied the substantial heterogeneity of the intrinsic proteins. The particle' shape varies from spherical (7–11 nm diameter; Fig. 3A, C, E) to rectangular (a = 15±1.6 and b = 9±0.8 nm, mean ± SD, n = 28; orange, Figs. 2B and 3A–C, E). While the spherical particles resemble the archetypical “bumps” of intrinsic proteins, the dimensions and shape of the rectangular particles (orange, Figs. 2B and 3A–C, E) matched the envelopes of the x-ray model of the AMPA-sensitive homotetrameric rat GluR2 receptor [30].

The density of rectangular particles in the entire spine was 72±10/ µm^2^ (mean ± SD, n = 7), which predicts a range of ∼196 to ∼246 putative AMPA-sensitive receptors per dendritic spine (∼3 µm^2^). They populated both the PSDs and the “non-synaptic” regions of the spine plasma membrane. In the E face clusters (i.e. the PSDs), their number varied from 0 to 64. This means that the largest number of putative receptor particles was only 30–40% of the total number of particles in the PSD (compare Fig. 3C and E). In the PSD, the putative receptors intermingled with the spherical “bumps” instead of self-associating into ordered patches. In “non-synaptic” regions of the post-synaptic membrane, these putative receptors formed bands alongside the major axis of the spine (rectangle, Fig. 3A and orange particles, 3B and E). In summary, our observations of the post-synaptic plasma membrane facing the active zone do not support the idea of a preferential and permanent distribution of neurotransmitter receptors.

### C. Vesicular Pools

Several schemes have been introduced to classify the synaptic vesicles according to functional properties (e.g. readily releasable and/or reserve pools) or to conformational states in the proteins of the trans-SNARE fusion complex (e.g. tethered, docked, primed, and/or super-primed). In this paper, we classify the synaptic vesicles according to their association with the active zone. We tentatively equate the smaller pool associated with the active zone. For descriptive purposes, we equate the vesicles associated with the active zone with the “readily releasable” vesicular pool, and all other vesicles as the “reserve” pool [15]–[17].

Independent of the association with the active zone, all synaptic vesicles have their proteins clustered at the poles of the phospholipid bilayer spheres (yellow, Figs. 2B, lower inset 4A). In one pole, the proteins form square domains ∼20 nm side that connect the vesicle to the cytoskeleton (see later). In the other pole, called the Z-pole, filaments measuring 14–16 nm in length and 4–5 nm in diameter are associated with the surface of the vesicle or protrude into the cytoplasm (large circle and lower inset, Fig. 4A). When the fracture plane exposes the “true” (un-fractured) surface of the Z-pole, the filaments formed pentagonal figures (small circle, Fig. 4B and cyan, Fig. 4C–D). In these pentagons, the free end of the filaments points either toward the vesicle's equator or straight upward, making the Z-pole resemble the head of the Gorgon Medusa from Greek mythology.

Even low magnification views of the interface indicate that synaptic vesicle docking involves the association of the Z-pole with the plasma membrane (P_pre_, E_pre_ in Figs. 2C, 4A–B; circles, Fig. 4A–B, cyan, 4C). To study this docking process, we selected cross-fractures because they show simultaneously views of the plasma membrane and the synaptic vesicle. These cross-fractures provided four distinct configurations, including the concave and convex hemispheres of the synaptic vesicles, and the P or E faces of the plasma membranes. Additionally, these configurations should face the E or the P face of the PSDs or “non-synaptic” regions of the spine plasma membrane (E_post_, middle red arrow, Figs. 2B; inset 2C). While the process of simultaneous tracking of fracture faces of the active zone (P_pre_, E_pre_), the synaptic vesicles, and the plasma membrane of the post-synaptic spine (E_post_ or P_post_), was often difficult, it guaranteed that the vesicles were involved in exocytosis rather than representing “reserve” vesicles that happen to be in the vicinity of the active zone.

**Figure 5:**
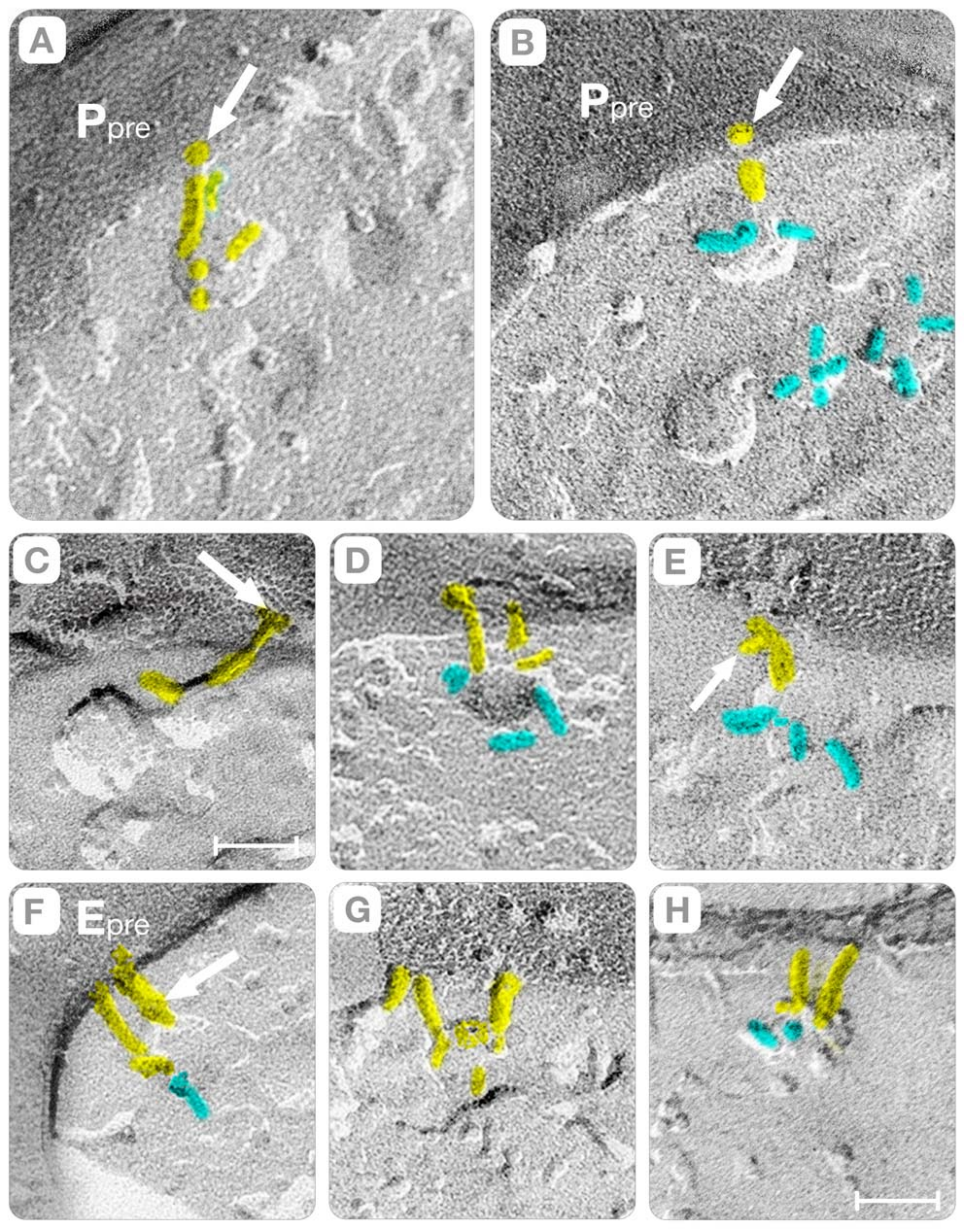
“Rods” Connect Vesicles to the Active Zone. To simplify the description, we show cross-fractures where the plasma membrane and the vesicle are imaged simultaneously. Moreover, the filaments extending from the vesicle into the cytoplasm are colored cyan and the “rods” connecting the vesicle to the active zone yellow. Panels **A–B** show synaptic vesicles connected to the plasma membrane (P_pre_) by rods oriented perpendicular to the plane of the membrane. Rod lengths are ∼20 nm in **A** and ∼15 nm in **B**. Independent of their length, however, the intra-membranous particles pointed by the arrows indicate that both rods transverse the plasma membrane at the ends. Panel **C** shows a vesicle connected to the active zone by rods ∼26 nm in length. A distinct kink in the mid-section, and small particles associated with the end that transverse the membrane (arrow), characterizes this rod. Panel **D** shows a vesicle connected to the E face of the active zone by two parallel rods (yellow). Panel **E** shows a rod connecting the vesicle to the P face of the plasma membrane. This rod has a domain protruding side ways into the cytoplasm (arrow). Panels **F** and **H** show synaptic vesicles connected by two parallel rods to the E face of the active zone. The arrow in **F** points to a distinct particle in the mid-section of the rod. Bars: Panels **A–C** = 45 nm; panels **C–H** = 35 nm.

**Figure 6:**
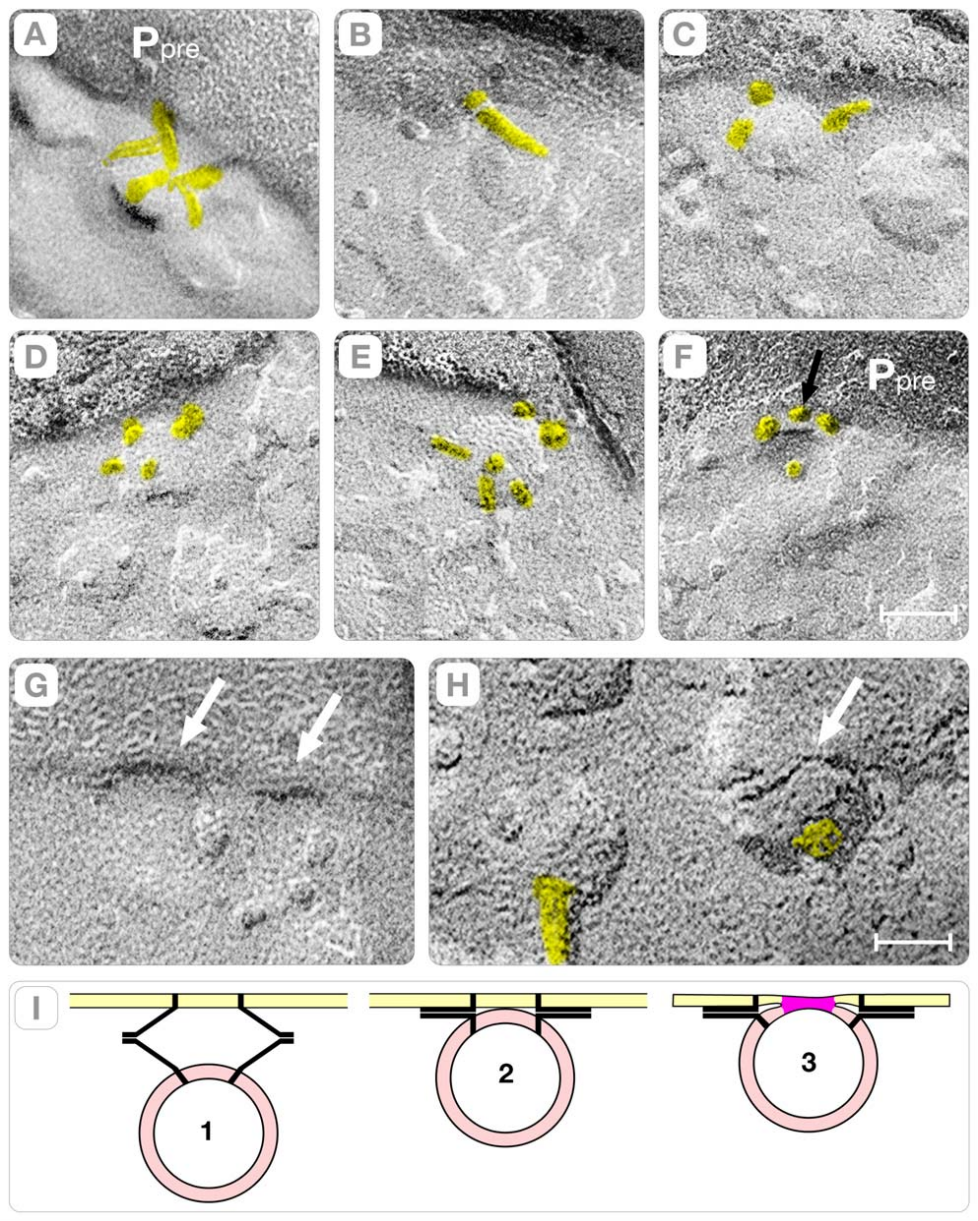
Vesicle Hemi-Fusion. In contrast to Figure 5, the rods connecting the synaptic vesicles to the active zone (yellow) are either tilted or oriented parallel to the plasma membrane. Panels **A–C** show the rods tilted at increasing angles with respect to the active zone (P_pre_). Panels **D–F**, the rods appear as particles sandwiched in the space between the vesicular and the plasma membrane (black arrow). Panels **G–H** show three synaptic vesicles in intimate contact with the membrane of the active zone (white arrows). The single ∼2nm in height fracture step (arrows) indicates that at the region of contact the vesicular and plasma membranes have hemi-fused. We note that the replicas are from rapidly frozen cortices without glutaraldehyde/osmium treatments. Bars: **A–F** = 35 nm, **H–G** = 25 nm. Panel **I** illustrates steps in the process association of synaptic vesicles to the plasma membrane. In this cartoon, the association occurs with the rods oriented parallel to the active zone. The degree of hybridization of the rods con (1 and 2) governs the distance separating the plasma membrane (yellow) from the synaptic vesicle (pink). In the final step (3), a single hemi-fused membrane (red) separates the lumen of the vesicle from the extra-cellular space.

The analysis of the cross-fractures indicated that protein complexes shaped as “rods” link synaptic vesicles to the membrane of the active zone (yellow, Figs. 5 and 6). These “connector rods” vary in length; their ends penetrate the plasma membrane (arrows, Fig. 5A–B) and have kinks in their midsection (arrows, Fig. 5E–F). The rod's length varies from 14 to 26 nm (yellow, Fig. 5A–H) and the midsection kinks often appeared as particles (arrows, Fig. 5E–F). At the point of contact with the plasma membrane, the “connector rod” exhibits a particle that reveals the trans-membranous nature of the end terminal domain (arrows, Fig. 5A–B). While the exact biochemical composition of the connector rod remains to be determined, the transmembrane nature of its end domain likely represents the trans-SNARE complex in its natural synaptic environment [12]–[14].

While the dimensions and trans-membranous nature of the “connector rods” rationalize how the synaptic vesicles associate with the plasma membrane, the opening of “fusion pores” requires the physical contact between the opposing membranes. In the cortical neuropil, two independent mechanisms transport the synaptic vesicles to the plasma membrane. In one mechanism, the aforementioned “connector rods” tilt their long axis until they become parallel to the plasma membrane (yellow, Fig. 6A–C). In this orientation, the “connector rods” appear as particles sandwiched between the concave or convex hemispheres and the P or E face of the plasma membrane (black arrow, Fig. 6C–F). Since the parallel orientation of “rods” does not specify the point of contact of the vesicle with the active zone, it agrees with the conventional “random” vesicle-fusion path as enunciated by the SNARE hypothesis [12]–[14].

**Figure 7:**
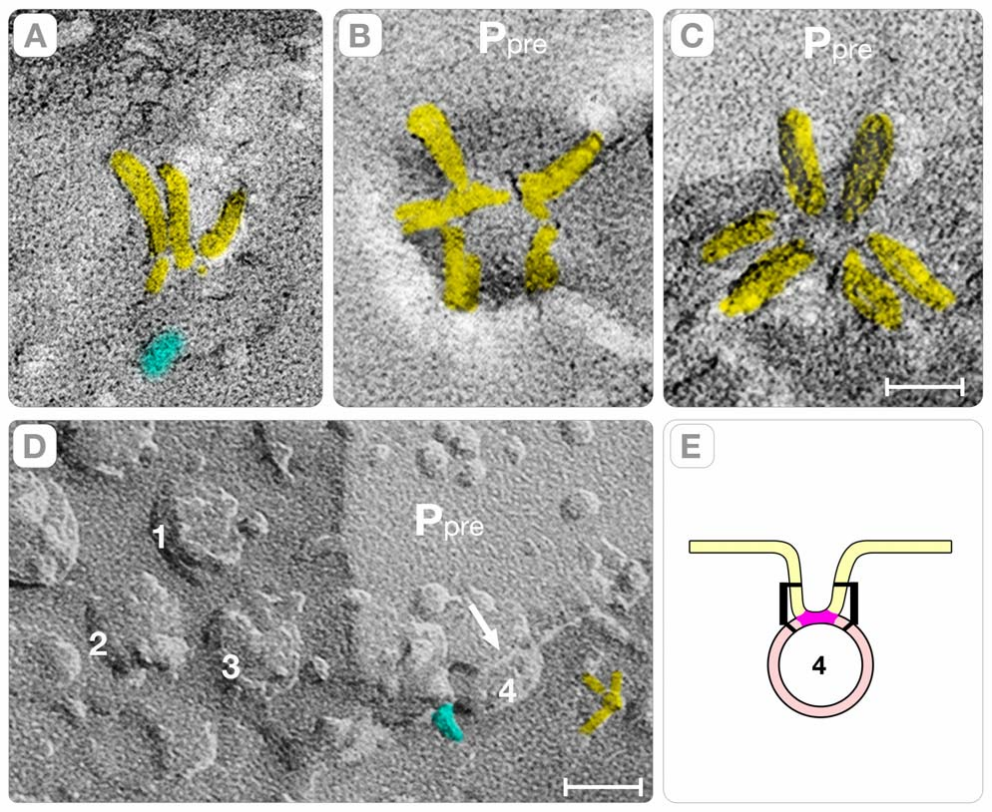
The Synaptic Vesicle/Indentation Complex. Panel **A** shows a lateral view of an indentation with three rods associated with its wall (yellow) and one protruding into the cytoplasm (cyan). Panel **B** is a top view of the plasma membrane (P_pre_) indicating that a complex arrangement of rods at the indentation's base attaches the synaptic vesicle to the active zone. Panel **C** shows the interface between the plasma membrane (P_pre_) and the cytoplasm. Several rods protruding from the Z-pole of the synaptic vesicle (yellow) connect the vesicle to the plasma membrane. Panel **D** shows the difference between synaptic vesicles in the cytoplasm (#s 1 to 3) and a vesicle that is hemi-fused to the plasma membrane (#4). The vesicles in the cytoplasm (“reserve”) are separated from the plasma membrane (P_pre_) by sizable aqueous spaces. The hemi-fused vesicle (#4) is identified by a single 2 nm height step at the region of contact with the plasma membrane (arrow). Note also several large intra-membranous particles placed in close proximity to the hemi-fused membrane. Bars: **A–C** = 25 nm, **D** = 40 nm. Panel **E** is an illustration of the proposed fusion path for a synaptic vesicle hemi-fused at the base of indentations. In this fusion path, vesicles must be connected to the active zone by rods oriented perpendicular to the plane of the plasma membrane.

A novel outcome of this study is that synaptic vesicles fusion also occurs with the hybrid “rods” oriented perpendicular to the plasma membrane (Figs. 7A–C). In principle, due to this orientation the vesicles should be separated from the active zone by aqueous gaps of thickness equal to the length of the “connector rod.” Instead, we found that elliptical depressions of the plasma membrane (called indentations) bring the synaptic vesicle into physical contact with the active zone (arrow, Fig. 7D). As illustrated in the diagram of Fig. 7E, the perpendicularly oriented “rod-shaped” macromolecules associate with the wall of the indentation and the synaptic vesicle hemi-fuses at its base. Since the perpendicular orientation of the “connector rods” creates a “primary curvature” of the plasma membrane at the base of which the synaptic vesicles will fuse, we call this mechanism the “deterministic” vesicle-fusion path (see also, Section D).

Independent on whether docking involves “rod” tilting or plasma membrane bending; a single membrane comprises the region of contact between the synaptic vesicle and the plasma membrane. In our study, the region of contact was identified because a single ∼2 nm height fracture step intervenes between the lumen of the vesicle and the extra-cellular space (white arrows, Figs. 6G–H and 7D). The aforementioned fracture pattern is consistent solely with “hemi-fusion,” which involves merging the external leaflet of the plasma membrane with the luminal leaflet of the synaptic vesicle at the contact region [25], [31]–[32]. Thus, our observations of rapidly frozen cortical neuropils without glutaraldehyde cross-linking are consistent with the concept that secretion of neurotransmitters involves the fusion-through-hemi-fusion hypothesis [33]. It is also important to underscore that vesicles at the base of the indentations are committed to exocytosis since hemi-fusion precedes the opening of the fusion pore.

### D. Indentations and Gutters at the Active Zone

**Figure 8:**
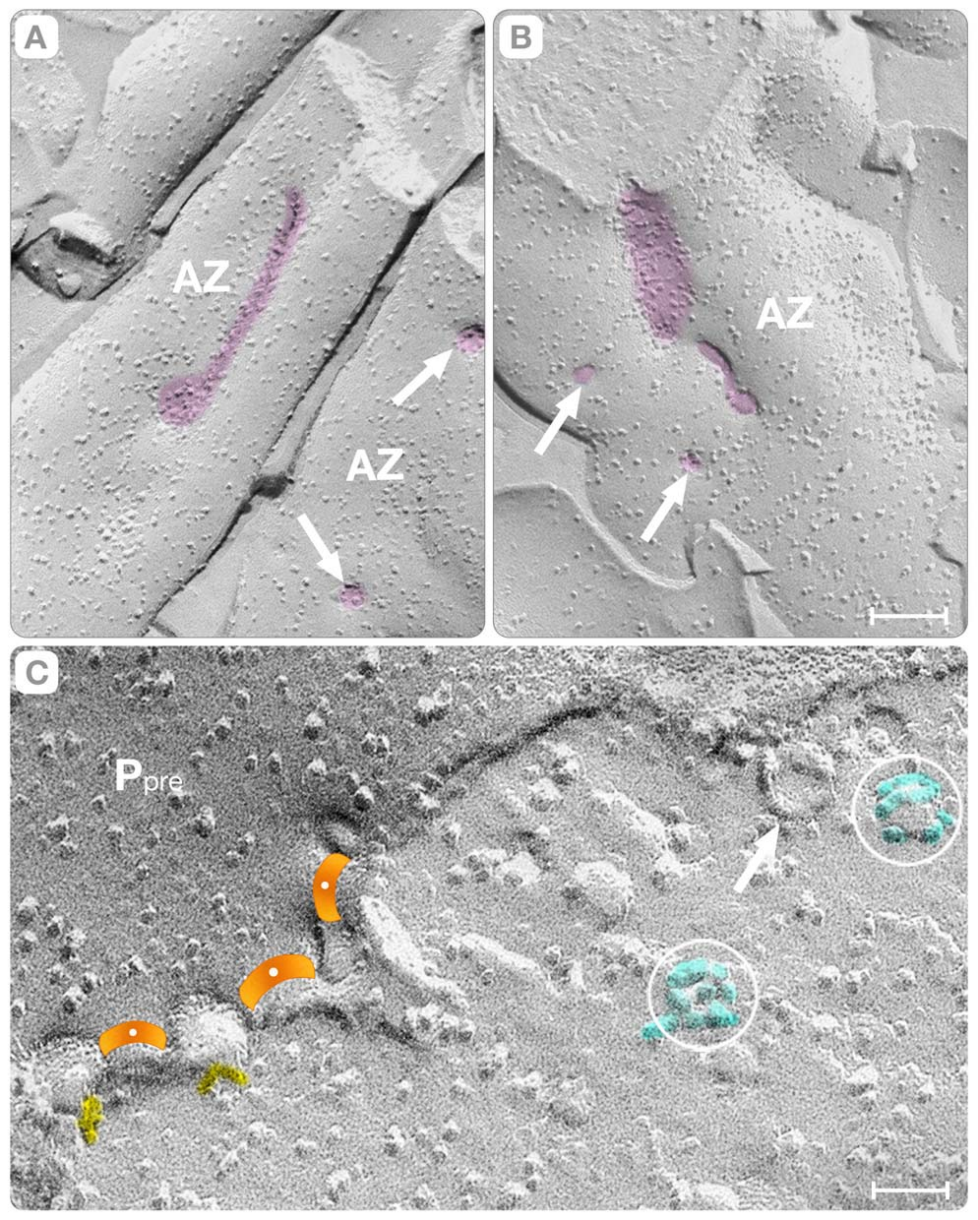
Marking the Active Zone. Panels **A-B** show the distribution of the intrinsic proteins, indentations (arrows) and gutters (magenta) in the active zones (AZ) of two contiguous axonal terminals. Panel **C** shows a gutter cross-fractured at the interface between the plasma membrane and the cytoplasm of the axon. The gutter has a file with four indentations, arranged in a file, spaced ∼80 nm center-to-center. In two of these indentations, short filaments protruding into the cytoplasm indicate vesicles hemi-fused at the base (yellow). The membrane between the indentations curves upward (orange bands). The white dots indicate “saddle” points where this positive curvature intersects the negative curvature of the gutter (see later). The white arrow points to a vesicle at the base of an indentation of the plasma membrane. In the cytoplasm, the fracture plane exposes two synaptic vesicles of the “reserve” pool (circles). The proteins are colored cyan to highlight their domain distribution on the surface of the vesicles. Bars: **A–B** = 0.14 µm, **C** = 40 nm.

**Figure 9:**
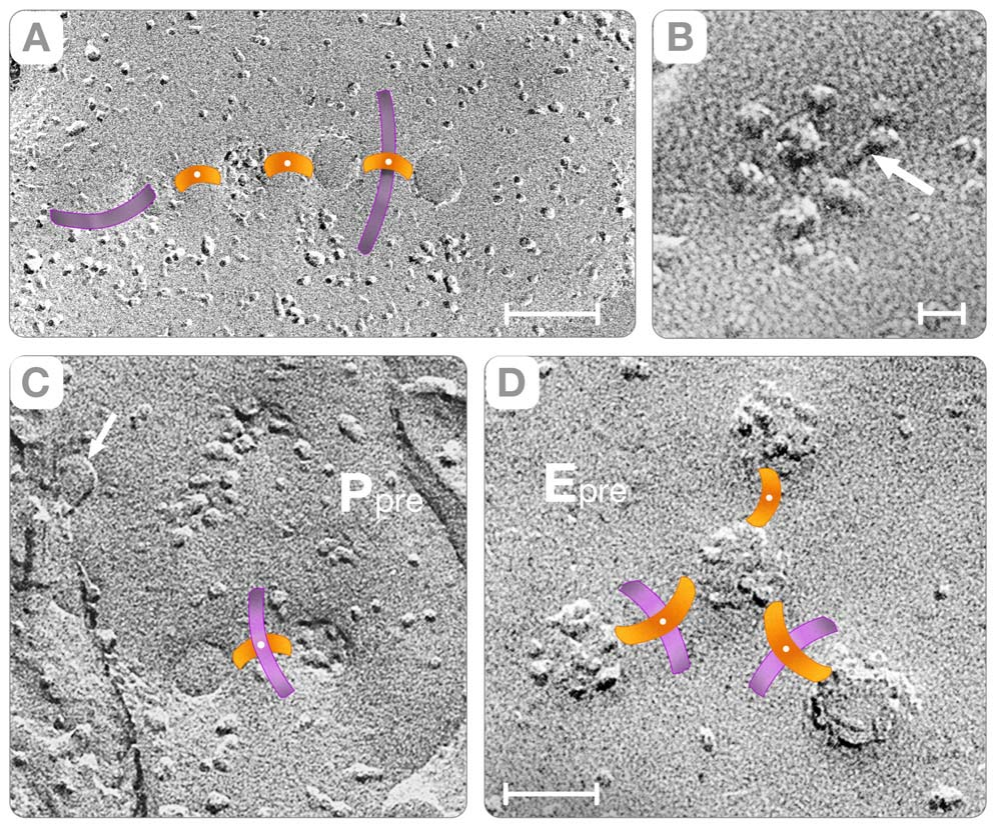
Curvatures of the Membrane of the Active Zone. Panel **A** shows a file with four indentations in the active zone of a cortical synapse. Upward directed membrane curvatures (orange bands) separate the indentations in the file. Three indentations have smooth walls, and one indentation has a cluster of intra-membranous particles. Panel **B** shows a higher magnification view of a cluster of intra-membrane particles at the bottom of some indentations. The particles are arranged in a ring around a larger (∼10nm diameter) central intra-membranous particle. The arrow points to an elongated particle in the ring. Panels **C** **and** **D** illustrate the two intersecting curvatures forming the gutters, called “primary” (magenta bands) and “secondary” (orange bands). In the P face, the primary curvature is directed downward and the secondary curvature upward (magenta bands, panels **C–D**). In the E face, the complementary that must exist between fracture faces inverts the signs of the curvatures: the primary curvature is upward and the secondary curvature downward (orange bands, panels **A, C–D**). The primary and secondary curvatures intersect in “saddle” (inflection) points because they have orthogonal directions and opposite signs (white dots, **A, C–D**). The saddle points are stationary and consequently the fracturing process does not affect their location. Bars: **A** and **C** = 80 nm; **B** = 10 nm; **D** = 40 nm.

The indentations are sharp crooks of the plasma membrane that bridge the space between the synaptic vesicle and the membrane of the active zone. In *en face* fractures, the indentations appear as elliptical depressions on the P face (P_pre_, arrows in Figs. 8A–B, and Fig. 9A, C) and complementary elevations on the E face (E_pre_, Fig. 9D). In the active zone, indentations exist either in isolation (arrows, Figs.8A, B) or arranged in files in which they are spaced 75–85 nm center-to-center apart (Fig. 8C and 9A). By examining stereo-pairs of synapses, we found that isolated indentations measured 35–45 nm in depth and are comprised of a single, primary curvature of the plasma membrane (magenta bands, Fig. 9C, D). Synapses with files of indentations revealed a secondary curvature, which intervenes between the evenly spaced indentations (orange bands, Fig. 8C, 9A,C–D). The secondary curvature is shallower than the primary curvature (15–25 nm in height) and bends the plasma membrane toward the extra-cellular cleft (upward) in the P face and toward the cytoplasm (downward) in the complementary E face (orange bands, Figs. 8C, 9A, C–D). Since the primary and the secondary plasma membrane curvatures exhibit orthogonal directions and reverse signs, they intersect at stationary inflection points, called “saddle” points (white dots, Figs. 8C and 9A, C–D). Interestingly, the arrangement of vesicles in files ensues the formation of gutters (or trenches) in the membrane of the active zone (magenta, Figs. 9A, C, and Figs. 8A–B) that increase the volume of the synaptic cleft. We estimated that the gutter created by a file with three evenly spaced indentations (80 nm×40 nm×240 nm) increases the volume of the extra-cellular cleft by ∼12–15% (∼1×10^−18^ liters).

**Figure 10:**
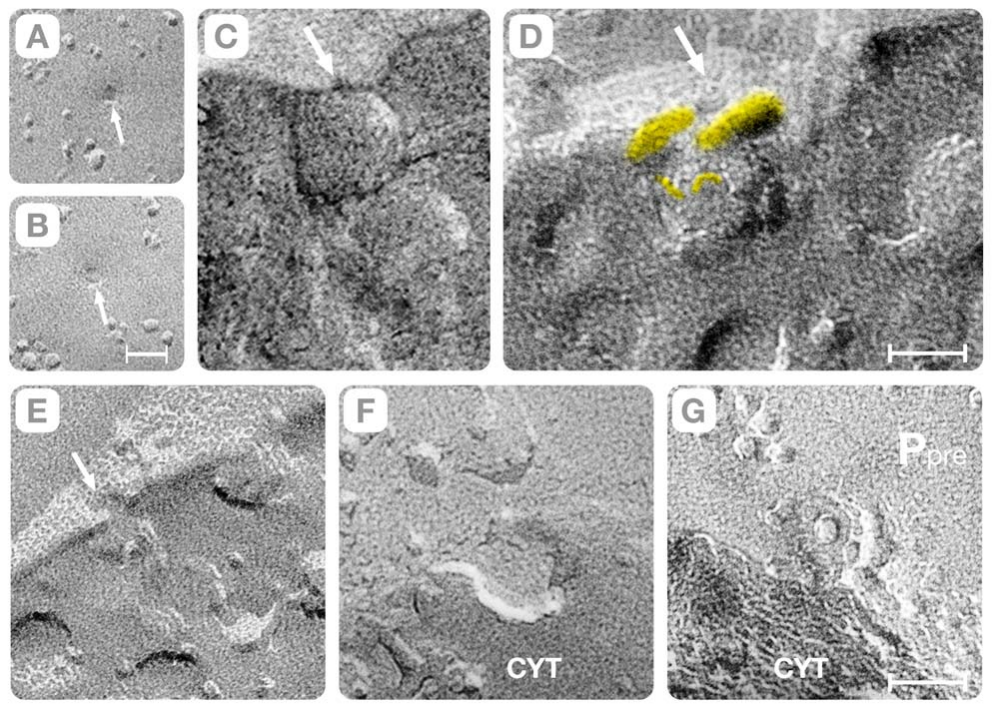
The Fusion Pore. Panels **A & B** show circular “holes” of the P face that represent the fusion pores (arrows). They were identified as fusion pores because occupied depressions of the plasma membrane and their shadow is the “reverse” from that of the intra-membranous particles (i.e. shadow in front, and metal accumulation in the back). Panels **C and D** are cross-fractures of the active zone indicating that the “holes” in the P face connect the lumen of synaptic vesicles to the extra-cellular cleft (arrows). Panels **E–G** illustrate the expansion of the hole (arrow, panel **E**) into fully fused synaptic vesicles (“omega” figures). Panel **G** is a top view of a cave to show the intrinsic proteins of a vesicle fused with the active zone. *Cyt* indicates the cytoplasm. Bars: **A–B** = 15 nm, **C–D** = 25 nm, **E–G** = 35 nm.

We also classified the indentations depending on whether a synaptic vesicle hemi-fuses at its base. Indentations with hemi-fused synaptic vesicles have elliptical shapes with semi-axes measuring a = 25±4 nm (n = 55) and b = 30±6 nm (n = 55) and depth = 33±3.5 nm (n = 55). They contain few intra-membranous particles (0–3) often placed at the junction between the vertical walls and the slightly elevated base (Figs. 9A, C). Indentations without hemi-fused synaptic vesicles at the base have also elliptical overall shapes with semi-axes measuring a = 50±10 nm (n = 38) and b = 60±11 nm (n = 38) and depth = 38±3.5 nm (n = 25). They contain clusters of up to 12 intra-membranous particles that are arranged in rings around a larger central particle (Figs. 9B, 10G). They might correspond to the so-called “omega” figures seen in thin sections of glutaraldehyde-fixed cortices.

### E. Fusion Pores

The fusion pores are discontinuities of the plasma membrane that connect the lumen of synaptic vesicles to the extra-cellular cleft. In freeze-fracture replicas, the discontinuities appear as “holes” in the P face and elevations in the E face. The P face “holes” are distinguished from regular intra-membranous particles because the shadows change location from the back to the front of the discontinuity (shadow “reversal”). Using this shadow reversal phenomenon, we identified the smallest fusion pore as a 3–5 nm in diameter “hole” (arrows, Fig 10A–B). A pore of this dimension is within the resolution expected from metal replicas measuring 1.2–1.3 nm in thickness [34].

In order to compare the pores of the vesicles comprising the conventional (“random”) and novel (“deterministic”) fusion paths, we relied once again in the examination of cross-fractures where the plasma membrane and the hemi-fused synaptic vesicle are imaged simultaneously. In both vesicle-fusion paths, the pores occupy the membrane that separates the vesicle's lumen from the extra-cellular space (arrows in Figs. 10C [deterministic] and 10D [random]). This observation is important because the fusion pore traverses a single membrane; an observation that contradicts models predicting that fusion pores somehow transverses two intimately apposed membranes [14].

The 3–5 nm diameter pores expanded rapidly into larger indentations that appear as “caves” of the plasma membrane, which diameter varied from ∼20 nm to ∼60 nm (arrows, Fig. 10E and F–G). The morphology of these caves depends on the path followed by the fracture plane. When passing through the walls, the fracture plane exposes clusters of up to twelve intra-membranous particles representing the integral membrane proteins of the vesicle that have fused at the active zone (Figs. 9A–B, 10G). At a later step, these rings of intra-membranous particles appeared as clusters intermingled with the intrinsic proteins of the membrane of the active zone.

### F. Simulations

**Figure 11:**
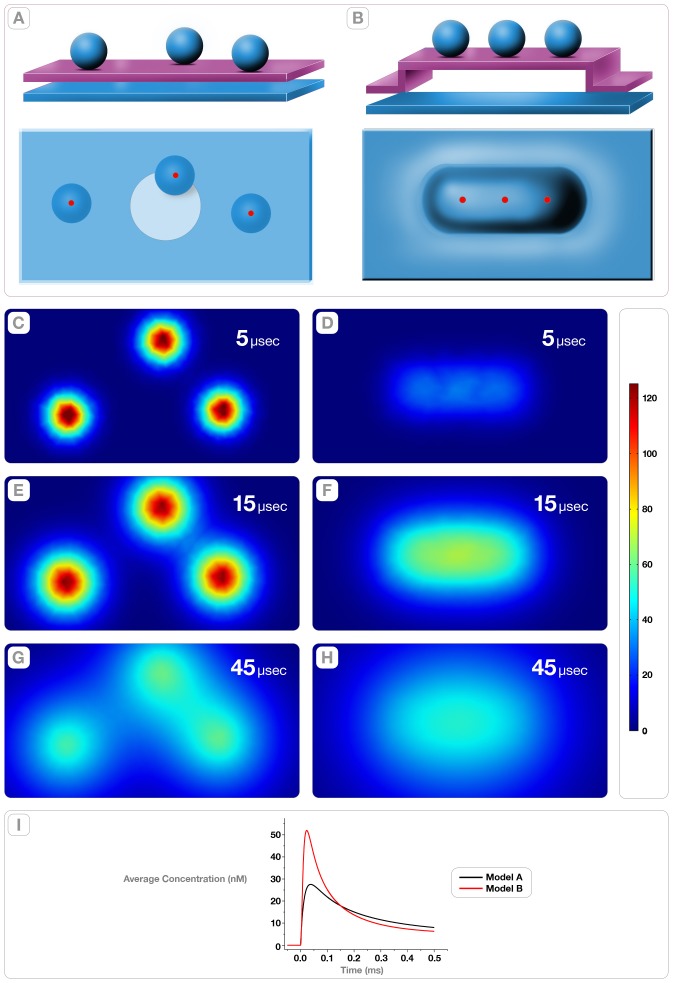
Diffusional Model Simulations of Release into the Post-Synaptic Spine. Panel **A** shows the conventional “random” vesicle-fusion path (Model A) constructed with the following structural parameters: synaptic cleft  = 30 nm, area of the active zone  = 1.65×10^5^ nm^2^, diameter of the vesicle  = 44 nm. The top figure shows the side view of the synapse and the bottom figure shows the external surface of the active zone. The red circles represent 4 nm diameter fusion pores and the larger white circle (100 nm diameter) a region in the post-synaptic membrane used to calculate the *average neurotransmitter concentration*. Panel **B** shows the “deterministic” vesicle-fusion model (Model B). A key difference between models A and B is the inclusion in the latter of a gutter in the active zone with dimensions a = 250 nm, b = 80 and depth  = 40 nm. All other dimensions were as in model A. All other panels show the neurotransmitter concentration domains at the post-synaptic that result from the simulations of model A (**C, E, G**) and model B (**D, F, H**). The simulation consisted in the calculation of neurotransmitter concentrations (after the simultaneous opening three vesicles loaded with 1.5 mM of neurotransmitter) computed at 5, 15, and 45 µs for models A and B. The results were calculated for each model by solving the 3D diffusion equation using a finite element numerical solver (Multiphysics Simulation Environment, COMSOL ver. 4.3.2). The pseudo color scale represents the neurotransmitter concentration (in nM) at the external surface of the post-synaptic spine. Panel **I** is a plot of the change in average neurotransmitter concentration over time (for both models A and B) in the region enclosed in the white circle as illustrated in panel **A**. The black line corresponds to the calculations in model A and the red line to those of model B. The peak average concentration is 28 nM (reached at 39 µs) for model A and 52 nM (reached at 22 µs) for model B.

To contrast the functional implications afforded by the synaptic vesicles' opening at the base of gutters (or indentations), as predicted by the novel (deterministic) fusion path, with those of conventional (random) opening, we simulated the neurotransmitter concentration domains on the spine plasma membrane (Fig. 11 A–B). In both simulations, three vesicles of 44 nm in diameter and loaded with 1.5 mM of neurotransmitter (∼2,000 molecules; Takamori et al., 2006) fused on an active zone measuring a = 550 nm and b = 300 nm. In both paths (Fig. 11A), the three vesicles open simultaneously into the extra-cellular synaptic cleft (30 nm width) via 4 nm diameter pores (red dots in lower panels, Fig. 11A–B). In the “deterministic” path (Fig. 11B), the three vesicles form a file where they are spaced 80 nm apart. We assumed that the three vesicles open simultaneously into a gutter that measured a = 250 nm, b = 80 nm and depth = 40 nm. In both models, the change in the *average concentration* of neurotransmitter was calculated over time in a circle of 100 nm in diameter at the center of the active zone (red and black lines, Fig. 11I).

The concentration of the neurotransmitter domains across the synaptic cleft (*c*) was solved with the 3D diffusion equation: 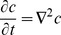



Where 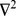 is the three-dimension Laplacian operator given by the divergence of the gradient as it applies to the neurotransmitter concentration in the synaptic cleft and *D*  = 1×10^−6^ cm^2^/s the diffusion coefficient. The solution relied on a finite element numerical solver (Multiphysics Simulation Environment, COMSOL ver. 4.3.2, Burlington, MA).

When vesicles fuse anywhere in the active zone (the “random” path, Fig. 11A), the neurotransmitter concentration domains on the spine plasma membrane peak 5 µs after opening and reach ∼125 nM (Fig. 11C). At longer times (15, and 45 µs), the domains expand and the neurotransmitter concentration at the spine plasma membrane decreases from ∼125nM to ∼60nM (Figs. 11E, G). However, when the vesicles fuse within gutters (the “deterministic” path, Fig. 11B), a shallower and broader neurotransmitter concentration domain focuses onto a single region on the spine plasma membrane (Fig. 11D). The domain peaks later (15 instead of 5 µs), reaches a lower concentration of neurotransmitter (∼70 instead of ∼125 nM) but it decays slowly to ∼60 nM at 45 µs.

Figure 11I compares the time course of the *average concentration* (in nM) of the 100 nm disk centered at the active zone for A (black line) and B (red line). Paradoxically, the average neurotransmitter concentration at the center of the active zone (100 nm diameter disk, lower panel in Fig. 11A) reaches a higher value (∼52 nM) in the “deterministic” instead of the “random” vesicle-fusion path (∼28 nM). The simplest explanation is that forcing the vesicles to open within gutters focuses the neurotransmitter domains into a broad region, which make the “deterministic” path better suited for stimulating the receptors in the “non-synaptic” regions of the plasma membrane of the post-synaptic dendritic spines.

## Discussion

Our study provides evidence that “rod-shaped” macromolecules that are oriented perpendicularly to the plasma membrane connect synaptic vesicles to the active zone in the rat cerebral cortex. A major consequence of this perpendicular orientation is the bending of the plasma membrane into curvatures with opposite sign that intersect at stationary “saddle” points. Since vesicle openings occur preferentially within these curved regions (i.e. trenches or gutters), they define a novel fusion path called here “deterministic.”

At the crux of the “deterministic” path is the mechanism by which synaptic vesicles attain physical contact with the membrane of the active zone. Current models propose that membrane contact involves the formation of the *trans*-SNARE complex by the hybridization of alpha helices from Q-SNAREs (i.e. syntaxin-1 and SNAP-25) and a R-SNARE (synaptobrevin) in a parallel configuration [12]–[14]. At the region of contact, the parallel *trans*-SNARE complex docks and fuses synaptic vesicles anywhere on flat regions of the active zone (i.e. randomly). Our study did identify synaptic vesicles with “connector rods” oriented parallel to the plasma membrane, which supports this conventional vesicle-fusion path. Nevertheless, the majority of the active zones imaged in this study have vesicles hemi-fused at the base of indentations that are arranged in files and spaced 75–80 nm center-to-center apart (Figs. 8C, 9A&D). This file arrangement of hemi-fused vesicles does not fit with the conventional “random” fusion path currently proposed by the SNARE hypothesis.

**Figure 12:**
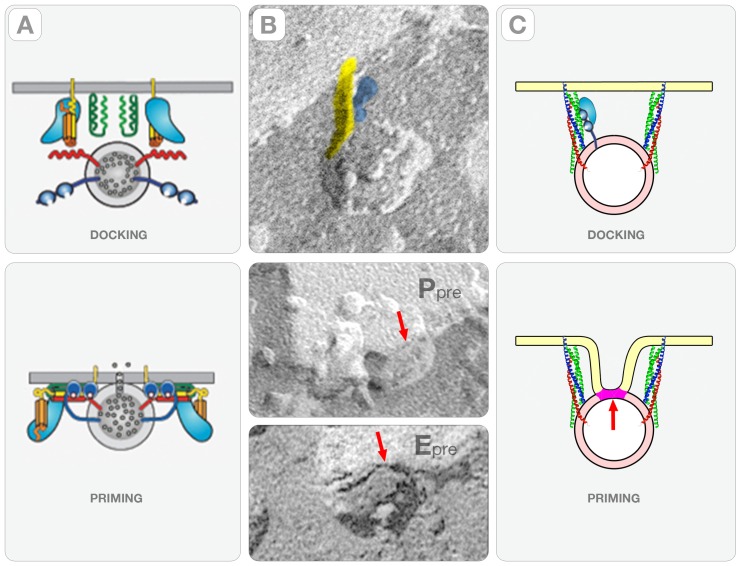
The “Deterministic” Synaptic Vesicle-Fusion Path. Panel **A** shows diagrams taken from a current model picturing the proteins and the conformational changes involved in the “docking” and “priming” steps of the neuronal exocytotic pathway [14]. The protein involved are: synaptobrevin (red) and synaptotagmin (blue) from the vesicle side and syntaxin, SNAP25 and Munc18 from the plasma membrane side. Panel **B** shows cross-fractures of the synaptic vesicle/plasma membrane interface in the synapses of the rat cerebral cortex. The upper panel shows a synaptic vesicle connected by a “rod” comprised of a straight (yellow) and a lobulated component (blue). The middle and the lower panels show the association of the vesicle to the P and the E faces of the plasma membrane. The red arrow points to the region of contact comprised of a single fracture step representing the hemi-fused membrane. Panel **C** shows a model of how a synaptic vesicle of the “deterministic” path could associate with the membrane of the active zone.

Thus, we propose a vesicle-fusion path in which “rod-shaped” macromolecules oriented perpendicular to the active zone (Fig. 12B) bends the plasma membrane into distinct indentations and gutters (Fig. 9) at the base of which vesicles dock and fuse. Evidence supporting the assignment of rod-shaped macromolecules as *trans*-SNARE complexes is that: a) rods occupy the vesicle/plasma membrane interface; b) their terminal ends span the phospholipids' bilayers of the plasma membrane (arrows, Fig. 5A–B); and c) their diameter and length can accommodate the four helices and the central cavity of the complex. Interestingly, the range of rod lengths (14–26 nm) that we measured in cerebral cortex synapses is wider than the (12–14 nm) estimated from measurements of purified SNARE proteins in solution [35] or the ∼10 nm reported in cryoelectron tomographic reconstruction of synaptosomes and organotypic slices [36]. Furthermore, and more importantly, the perpendicular configuration of the connector rods implies that the alpha helix of the R-SNARE synaptobrevin hybridizes in an antiparallel configuration when assembling the *trans-*SNARE bundle (as illustrated in Fig. 12B&C). Interestingly, this is in agreement with studies using single molecule fluorescence resonance energy transfer (FRET) of pre-assembled neuronal SNARE complexes showing that SNARE motives typically occur as a mixture of parallel and antiparallel configurations [37].

Another remarkable property of the deterministic path is the reproducible “marking” it introduces in the active zone in the form of sharp crooks (indentations) and shallow depressions (gutters) (Figs. 7C and 9A, C–D). By examining stereo-pairs of the active zones, we found that these “marks” result from the interplay of two curvatures of the plasma membrane, which we term “primary” and “secondary” (magenta and orange bands, Fig. 9A, C–D). The primary curvature, a direct consequence of the perpendicular orientation of the hybrid “rods” associated with the wall of the indentation, explains the individual synaptic vesicle/indentation complexes in the active zone (arrows, Fig. 7A–C). The secondary curvature, likely a reflection of the ∼60 nm diameter polyhedral cages that form the “pre-synaptic grid” in the active zone of cortical synapse, intervenes between the indentations in the file (orange bands, Figs. 8C, 9A, 9C–D). (The “pre-synaptic grid” is one of the most defining structures of the active zone in cortical synapses [2], [31]. Since the primary and secondary curvatures exhibit orthogonal directions and opposite signs, they intersect at stationary “saddle” points (white dots, Figs. 7C, 9A, C–D), which we propose to ultimately impose the long-range order of the hemi-fused vesicles of the “deterministic” vesicle fusion path.

Independent of whether synaptic vesicles follow the conventional “random” or the “deterministic” fusion path, at the region of contact the external leaflets merge, a process referred as hemi-fusion [33]. Synaptic vesicles hemi-fused with the active zone membrane were first recognized in conical 3D-reconstructions calculated from synapses in the rat cerebral cortex and ribbon synapses in the mouse retina, which were prepared by conventional thin sectioning methods [25], [31]–[32]. In contrast, synaptic vesicle hemi-fusion was not resolved in cryoelectron tomography of isolated synaptosomes and organotypic slices from rat hippocampus [36].

In this study, we examined cortices that were rapidly frozen without glutareldehyde cross-linking while using improved freeze-fracture methods. We identified the hemi-fused vesicles from the fracture steps that separate their lumen from the extra-cellular space at the synaptic cleft (arrows, Figs. 6G–H and 8D). An interface comprised of two closely apposed membranes results in two fracture steps (∼2nm height); instead, a hemi-fused membrane produces a single fracture step (lower panels, Fig. 12B–C). Thus, our observation single steps at the vesicle/plasma membrane interface is only consistent with the hypothesis that the fusion pore spans a single membrane, instead of the two anticipated in most models of neuronal exocytosis (Fig. 12A). Furthermore, our study guaranties that synaptic vesicles at the base of the indentations are committed to exocytosis because hemi-fusion precedes fusion pore formation.

We used computer simulations to compare the neurotransmitter concentration domains induced on the post-synaptic spines by synaptic vesicles opening on flat or at the base of gutters in the active zone (Fig. 11A–I). The idea behind these simulations was to explore the theoretical implications that the bending of the plasma membrane at the active zone has on neurotransmitter release since this region is currently inaccessible to experimentation. Without this modeling, the paper would have been purely descriptive. When the vesicles open on flat regions, the computation indicates independent domains that reached high concentration of neurotransmitters (∼125 nM). In contrast, when the vesicles open within the gutters defined by stationary “saddle” points, the computation indicates a single domain that peaks slowly and reaches lower concentration of neurotransmitters (∼60 nM). Paradoxically, the average concentration of neurotransmitters in these domains (whiter circle, lower panel Fig. 11A) is higher when vesicles open within gutters (52 nM versus 28 nM, Fig. 11I). This means that the gutters are low-pass filters that focus release over extensive regions of the spine plasma membrane (Fig. 11, D, F and H). We hypothesize that this property of the “deterministic” vesicle-fusion path is better suited to stimulate the neurotransmitter receptors located in the “non-synaptic” regions of the spine plasma membrane.

Our observations impinge also upon the arrangement of the neuropil in the rat cerebral cortex, which has remained mostly undetermined for technical reasons (the “projection artifact”). In the pre-synaptic axons, we recognized a sizable enlargement (the “varicosity,” Fig. 2C) that increases the surface area (6X) and the volume (11X) of the terminal. The enlargement of the pre-synaptic axonal terminal matches post-synaptic dendrites with “spine apparatuses” and “coated” vesicles (Fig. 1B). It seems manifest that the confluence of specialized organelles in the pre- and post-synaptic terminals underlines the distinctiveness of the structure of the synapses in the neuropil of the cerebral cortex.

In conclusion, plasma membrane curvatures that result from the perpendicular orientations of “rod-shaped” macromolecules connecting vesicles to the active zone define a novel vesicle-fusion path and preconditions specific regions of the active zone to favor coordinated neurotransmitter releases in the rat cerebral cortex. We hypothesize that a simple change in the hybridization of the R-SNARE synaptobrevin from parallel to antiparallel swings synapses into this novel “deterministic” vesicle-fusion path.

## Methods

### Ethic Statement

The studies were carried out in strict accordance with the recommendations in the Guide for the Care and Use of Laboratory Animals of the National Institutes of Health. The protocols were approved by the Committee on Animal Care and Use Committee, known as the Chancellor's Animals Research Committee (ARC) at UCLA (ARC #1994-244-52). All surgery was performed under sodium pentobarbital anesthesia, and all efforts were made to minimize suffering.

### Animals

We used the cerebral cortices of 45 Sprague-Dawley rats between P15 and P24 of either sex. The brains were removed and immersed in cold (4°C) saline saturated with 95% O_2_ and 5% CO_2_. 100–150 um-thick slices were cut with a Vibrotome (TPI Leica VT-1000) in the coronal plane. The cerebral cortex was dissected from the dorsal surface of both hemispheres just posterior to the anterior commissure. These regions represent frontal associations and the motor neocortex. The subcortical white matter was dissected away from the six-layered cortex.

### Thin Sectioning

The method has been described in several studies [25], [31]-[32], [38]. Shortly, the dissected neocortices (1.3×0.75×0.1 mm) were immersed in solutions with 3% glutaraldehyde and 0.5% tannic acid in 0.2 M Na cacodylate buffer pH 7.3, for 2 hours at room temperature. After washing five times in 0.1 M Na cacodylate plus 10% sucrose, the dissected cortices were postfixed in 1% OsO4 prepared in the same buffer for 90 min. The cortices were then washed in 0.1 M Na acetate pH 5.5 and block-stained in 0.1 M Na acetate with 0.5% uranyl acetate for 12 hours at 4°C. The cortices were dehydrated in ethanol, passed through propylene oxide and infiltrated in mixtures of Epon 812 and propylene oxide. The samples were embedded in pure Epon 812 and cured in an oven at 60°C for 48 hours. Thin sections with gray-to-silver interference colors (55-80 nm thickness) were cut and deposited on 200 mesh grids coated with carbon only or on single-hole (∼600 µm diameter) grids coated with formvar and carbon. On the grids, the sections were floated on drops of 2% uranyl acetated for 10 min and lead citrate for 3 min. The exposed surface of the section was coated with a thin layer of carbon to reduce the “shrinkage” induced by radiation during imaging.

### Freeze-Fracture

#### Conventional Experimental Approach

In a first attempt, we used the conventional freeze fracture method in which small pieces of neocortices (<1 mm^2^×0.1 mm) were fixed by immersing them in solutions containing 3% glutaraldehyde in 0.2M cacodylate buffer pH 7.3 for 2 hours at room temperature. To avoid ice crystal formation, the pieces were later transferred to 20% glycerol in 0.2 M Na cacodylate buffer pH 7.3 and deposited on gold-coated specimens holders. Freezing involved plunging the holders first in liquid propane and then in liquid nitrogen. After transferring to a Balzers 400K Freeze-Fracture-Etch apparatus, the frozen cortices were fractured at −150°C and shadowed immediately with Pt-C at 45° and pure carbon at 90°. However, it is important to stress that *this conventional preparative protocol failed to image the structures connecting the vesicles to the active zone*.

#### Improved Experimental Approach

To study the connections between vesicles and active zones, it was necessary to: a) minimize “plastic deformation” during cleaving; b) correct for the angle of metal deposition; c) enhance “pure shadowing” while avoiding preferential nucleation (“decoration”) that results when metal interact with groups exposed on the fractured surfaces; d) have access to the JEOL RFD-9010CR-Freeze-Fracture-Etch apparatus; and e) protect the replicas from fragmentation.

Plastic deformation refers to the damage that occurs when the cleaving knife touches the tissues in the freeze-fracture apparatus. We reduced the effects of plastic deformation by fracturing rapidly frozen tissues without chemical crosslinking at −104°C instead of −150°C. The angle of deposition was an important parameter for improving the quality of the metal replicas. Shadowing at a fixed 45° angle induced long shadows that masked the internal structure of the intra-membranous particles representing intrinsic synaptic proteins and reduced the steadiness of the replicas during, tissue digestion, washing and handling. We reduced the shadow lengths by tilting the platinum-carbon electrode at 80° with respect to the fractured surface while simultaneously rotating and tilting the frozen tissue in the X, Y and Z directions during the carbon evaporation (i.e., the “multi-axes-shadowing”).

Pure shadowing reports the relief of structures exposed on the fractured surface. In contrast, “decoration” reflects the physico-chemical properties of the frozen surface, including the exposure of bulky amino acid side-chains and charged groups. The interplay of a series of experimental conditions, including the affinity of the metal to frozen tissue as well as the presence of ions, variations in the temperature and the vacuum conditions during evaporation determines whether “pure shadowing” or “decoration” dominate image formation. We found that shifting into “pure shadowing” mode of image formation required pre-coating the fractured surface with a thin layer (2s) of pure carbon and pulsing the deposition of platinum and carbon. These experimental manipulations enhanced pure shadowing and provided a better definition of intra-membranous particles and protein complexes.

In this study, cortices were rapidly frozen on gold-coated hats in a Life Cell CF100 unit without fixation and/or cryoprotection. The frozen cortices were stored in liquid nitrogen refrigerators and transferred into a JEOL RFD-9010CR-Freeze-Fracture-Etch apparatus. The cortices were fractured with a liquid nitrogen cooled knife at -104°C and 1×10^−7^ mbar partial vacuum pressure. After fracturing, the specimen was re-cooled back to −175°C, pre-coated with a layer of pure carbon deposited for two seconds at 90°. The procedure was followed by depositing platinum-carbon at 80° in two four seconds pulses and one ten seconds pulse with pure carbon at 90°. During the carbon deposition, the specimen was rotated simultaneously along the x, y and z directions (“multi-axes” shadowing).

After removal from the freeze-fracture apparatus, the replicated frozen cortices still on the specimen holders were coated with a drop of 0.5% collodion in amyl acetate. This step avoided the natural tendency to fragmentation that occurs during removal of the un-fractured tissue. The tissue was digested in 100% bleach and the replicas washed in distilled water and deposited either on single-hole grids coated with Formvar and carbon or on 200 mesh grids coated only with carbon. The layer of colloidon was removed by immersion in a solution of amyl acetate. The procedures outlined above produce replicas 1.2–1.3 nm in thickness comprised of four metal grains. This procedure has been used in previous studies [21]–[24], [34], [39]–[41].

#### Imaging, Sampling and Data Analysis

We used a Zeiss EM10C electron microscope operated at 80 or 100 kV. The thin sections and freeze-fracture replicas were imaged at 5,000× and 10,000× and synapses at 25,000× and 50,0000X. Images free from optical aberrations of synapses exhibiting the highest signal-to-noise ratios (thin sectioning) and frozen with minimum ice crystal damage were selected for further study. The criteria used for judging these attributes were: a) sphericity and constant diameter of synaptic vesicles; b) preservation of mitochondrial structure; and c) visualization of the bilayer structure (unit membrane) of the membranes pre- and post-synaptic terminal. Using this approach, we selected ∼800 negatives with ∼3,300 synapses from a database larger than 25,000 negatives. The selected negatives were printed and studied to choose individual synapses that were further digitized using a Nikon Super Coolscan 9000 microdensitometer and processed using the ImageJ software package. The analysis consisted in measuring: a) the areas of P and E faces of the active zone and the density of intra-membrane particles, b) the area of the post-synaptic densities and the number and density of particles on the E face, c) the dimensions of the particles in the post-synaptic domains, d) the “filaments” linking the synaptic vesicles to the active zone and the regions of hemi-fusion, e) the number and dimensions of the indentations in the active zone, the number of particles on the walls, and d) the synaptic cleft.

#### Stereo Pairs

We collected a limited number of stereo pairs (23) by imaging the same region in a replica tilted ±6° with respect to the viewing direction. Their analysis provided information about the directions and signs of the curvatures in the indentations and gutters of the active zone in cortical synapses and the identification of the saddle points (not shown).
